# Physiological and Pathological Researches

**Published:** 1837-10

**Authors:** 


### ART. VII.

*Untersuchungen zur Physiologie und Pathologie.* Von Dr. FRIEDRICH NASSE und DR. HERMANN NASSE. Erstes, Zweites und drittes Heft. —*Bonn*, 1835-6.

*Physiological and Pathological Researches.* By DR. FREDERICK NASSE and DR. HERMANN NASSE. Parts I. II. and III.—*Bonn*, 1835-6. 8vo. pp. 486.

THE work whose unpretending title we have just transcribed, is one of a class not uncommon on the continent, but sufficiently rare in this country. It may perhaps be difficult to account for the fact, (that it is a fact few will be disposed to deny,) that, though Britain has contributed her full share to the advance of the science of physiology, this share has been furnished by a small number of individuals whose discoveries have earned for them a lasting reputation; and that, in proportion to the number of well-educated practitioners which she possesses, very few are engaged in the pursuit of that most important and interesting branch of enquiry. Such a work as we have now before us, consisting of original researches of great value on many of the most obscure and complicated questions in the science, would indeed be a rarity in this country; for, though many valuable papers are scattered through our various Transactions and Journals, we know of few who make the pursuit of experimental physiology the employment even of their leisure hours, much less the business of their lives. We shall therefore devote more than ordinary space to the analysis of the Essays now before us, partly on account of their intrinsic value, and the improbability of their being presented to our readers under any other form; and partly in the hope of exciting some of our brethren in this country to a similar course of practical research.

The two Drs. Nasse of Bonn (father and son,) are already favorably known as experimental physiologists; and these researches will, we doubt not, considerably increase their reputation. The authors state it to be their intention to publish three or four numbers of these essays every year, chiefly on physiological questions, but with an occasional glance at pathological subjects. Of all those which have already appeared, it is our intention to present our readers with a full account; but, on the present occasion, our limits compel us to restrict our attention to two of the most important, by the oldest of the authors, Dr. Frederick Nasse.

I. *Observations and Experiments on the Functions of the Spinal Cord.*

The essay, of which we now proceed to give an account, is contained in the Second Part, and is on one of the most interesting subjects which have recently engaged the attention of physiologists. Although rather calculated to unsettle than to confirm opinions usually regarded, in this country at least, as well established, we cannot hesitate to allow considerable weight to the facts and arguments adduced by the author.

Although the views of Sir C. Bell on the separation of the motor and sensitive tracts of the spinal cord are very generally if not universally received in Britain, the number of experimental physiologists on the continent who have advocated or denied this separation, is pretty equally balanced. We shall not enter into the discussion of this important question for the present, as our object is to lay the facts and observations of the author before our readers, rather than the expression of our own opinions; but it must, we think, be admitted that a distinct separation of function, as relates to the fasciculi of the spinal cord, has not yet been established in such a manner as to preclude all further investigation.

The facts obtained by the observation of disease are not regarded by Dr. Nasse as favorable to the doctrine, that the anterior fasciculi are exclusively motive, and the posterior solely concerned in sensation. He mentions several cases in which compression or degeneration of the posterior part of the cord was unaccompanied by corresponding loss of sensation, whilst a similar change affecting the anterior fasciculi had destroyed the power of motion: some of these fell under his own notice; others are recorded by different authors* to whom he refers. He then goes on to observe, that with the exception of one not very accurately detailed, we have no well-authenticated cases of loss of sensibility and retention of the power of motion, coexisting with a morbid state of the posterior and natural state of the anterior fasciculi, although this state is more frequent than the reverse condition; and he gives the following as his conclusions from pathological phenomena.

"1. In the majority of cases in which lesion of motion had been present, degeneration of the anterior fasciculi of the cord was discovered.

"2. Sensation did not remain wholly undisturbed where the effects of degeneration or pressure had been limited to the anterior fasciculi. (A remarkable instance of complete loss of sensibility and of motion, resulting from the pressure of a tumour on the anterior column only, is related by Velpeau, Archives Générales de Medecine, Janv. 1835.)

"3. It has also occurred that, in complete paraplegia, the degeneration has been limited to the posterior fasciculi.

"4. There are cases on record in which very slight morbid change was found in one anterior fasciculus, while the other close to it was found considerably degenerated; yet the power of motion had been lost on the side in which the slight change occurred."

Experiments on animals furnish more accurate and satisfactory information on matters purely physiological† than on those which have also a

* Fodera, Journal Complementaire, Cahier 80. p. 269; Serres, Anatomie comparée du Cerveau, tom. ii. p. 221; Abercrombie on Diseases of the Brain and Spinal Cord, 2d Ed., pp. 362, 365, &c.

† We presume that the terms *mental* and *physiological* are used, by our author, as synonymous with the *sensorial* and *nervous* of Dr. W. Philip.—Rev.

mental relation; but they may be used, under certain restrictions, to illustrate the mental relations of the spinal cord. We shall state Dr. Nasse's views on this subject nearly in his own words, considering them highly deserving of attention.

"If we examine the relations which the anterior and posterior fasciculi of the cord bear to sensation, it would appear, first, that the posterior fasciculi are peculiarly sensible; and next, that the anterior fasciculi are evidently less sensible than the posterior. These points deserve further consideration.

"In making experiments on animals to ascertain the relations which the anterior and posterior fasciculi of the spinal cord bear to sensation, there is one source of error which appears equally unavoidable and irremediable. The gradations of sensibility are almost imperceptible; the shades are so delicately and so intimately blended that every attempt to determine the line of transition proves inadequate. There is a great deal of truth in an expression of Calmeil's—that it is much easier to appreciate a hemiparalysis of motion than a hemiparalysis of sensation. If the anterior fasciculi of the cord possess sensibility, but only in a slight degree, the mere opening of the vertebral canal and laying bare the cord, must cause such a degree of pain as would weaken or destroy the manifestations of sensibility in the anterior fasciculi. This has not been sufficiently attended to by experimenters. Again, the practice of first irritating the posterior fasciculi, and afterwards the anterior, must have had considerable effect in producing the same alteration. It is plain that, in this way, the relations which the anterior fasciculi bear to sensation must be greatly obscured; yet, with the exception of some few experiments, this has been the order of proceeding generally adopted.

"Another circumstance, weakening the inference from the experiments, is the very occurrence of violent convulsions in those muscles which are under the influence of the anterior fasciculi. Such violent muscular motions must obstruct, more or less, the manifestations of pain, when the anterior fasciculi are irritated. The hind legs of a rabbit being enveloped in such a way as to prevent motion, the spinal cord laid bare, and the anterior fasciculi irritated with a blunt needle, a shrinking of the parts above and below the irritated point was noticed, exactly resembling that which results from irritation of the posterior fasciculi; and in two of the cases in which this phenomenon did not occur, the laying bare of the cord had caused a more violent degree of hemorrhage than usual, and the manifestations of sensibility, even in the posterior fasciculi, were feebly expressed."

In the opinion of Dr. Nasse, therefore, experiments on the lower animals cannot be held as exclusively establishing the relations which the fasciculi of the cord bear to sensibility; and, in examining how far their connexion with muscular action has been established, he points out many circumstances which may prejudice the accuracy of the results usually relied on.

"In the first place, voluntary muscular motions should be distinguished from mere manifestations of irritability. Again, the laying bare of the cord must produce more or less disturbance of the motive faculty, and must particularly affect any capability of motion possessed by the posterior fasciculi. A rabbit, of the variety with long silky hair, was observed to become paralysed in its hind legs after this operation, although every precaution was taken to avoid shattering of the bones or disturbance of the nervous matter. The rough way, also, in which the anterior fasciculi are divided in most experiments cannot fail, in some degree, to stretch or press the posterior fasciculi, so as to affect the relations which the latter may bear to voluntary motion.

"A circumstance which also tends to throw additional obscurity on this subject is the difference of opinion as to the exact nature of the phenomena observed after division of the posterior fasciculi in the parts dependent on them for nervous influence. Some assert that this operation completely abolishes all voluntary power; others, on the contrary, state that, under the same circumstances, they have witnessed motions of these parts which had all the appearance of being voluntary. In a case of this description, a single well-established positive result is worth a host of negative ones. The results obtained by Rolando, Bellingeri, and Seubert on this point, are sufficiently known. In Calmeil's experiments on lambs, irritation of one of the posterior fasciculi produced, in almost every instance, motions of the corresponding extremity; and, after division of the anterior fasciculi, irritation of one of the hind legs, or its nerves, excited motions in both. There can be no doubt that the anterior fasciculi have a relation to voluntary motion; but it seems also probable that they possess some share of sensibility.

"If we sum up all the foregoing facts, derived as well from cases of disease as from experiments on animals, it appears to result that in man, and in the animals which approximate him, a division of the functions of the anterior and posterior fasciculi of the spinal cord is not yet satisfactorily proved."

We have given these statements of our author without comment; but, in order that they may not bring us back to the state of "glorious uncertainty" which so lately prevailed as to the functions of the nervous system, we beg to subjoin a few observations.

1. Although we agree with Dr. Nasse that there is still some difficulty as to the parts of the spinal cord appropriated to sensation and to voluntary motion, (which, indeed, is sufficiently obvious when we attend to the ascertained power of the spinal accessory nerve in exciting motion, and to its obvious origin *behind* the ligamentum denticulatum,) yet, when we consider that the result, both of experiments and of pathological observations on the *nerves of the face and eye*, has decidedly established the separate endowment of these nerves, as stated by Bell, we can scarcely doubt that a similar difference of endowment exists in the constituent filaments, both of the spinal nerves and of the spinal cord, however difficult it may be, in the more complex structure of these parts, to distinguish the precise filaments employed for their different purposes.

2. Dr. Nasse does not seem to be aware of the modification which the doctrine of Bell has undergone in consequence of his dissections, the results of which are recorded in the Philosophical Transactions for 1834 and 1835, which convinced him that the posterior roots of the spinal nerves arise from a portion of the *lateral* columns of the spinal cord descending from the crura cerebri, and decussating in the medulla oblongata;—not from the *posterior* columns which descend by the corpora restiformia from the cerebellum, and do not decussate.* This being so, we at once perceive that much disease of the posterior portion of the cord may be unattended with loss of sensation in the parts below.

3. In the preceding and some of the following statements, we think Dr. N. has not duly reflected on the important principles which we think fully established in pathology, and always to be kept in mind in attempting to draw physiological inferences from morbid appearances in the nervous system; viz. that nervous matter may undergo great changes of form, and even of apparent structure, if gradually effected, without losing vital power; and that the influence of disease or injury more rapidly affecting the nervous matter may extend to the alteration or suspension of the functions of parts connected with them, which have not themselves undergone any obvious disorganization.

4. In this paper two questions are involved, which ought to have been

* Bell on the Nerves, 3d Edition, pp. 218, 19.

kept distinct. One respects the functions of the anterior and posterior filaments of the spinal nerves; the other respects the functions of the anterior and posterior fasciculi of the cord. With respect to the first, it may be considered fully established that irritation of the posterior filaments of nerves, when separated from the cord, never produces motion; whereas, irritation of the anterior filaments, similarly separated, invariably produces motion; and that if, of one hind leg of a frog, all the anterior filaments of the nerves be divided, of the other hind leg all the posterior filaments, the first has lost all power of motion, the latter all sense of feeling. It follows, from these experiments of Müller on frogs, (animals which long survive the opening of the spinal canal, whose nerves long retain their sensibility, and whose roots of nerves for the posterior extremity run for such a considerable space in the canal separate, that no mistake can occur as to which are divided, and which are so thick that no one of them can be overlooked,) that Bell's theory with respect to the nerves is fully established.

But, with respect to the other question, the functions of the several regions of the cord, experiment has decided nothing absolutely; and pathological results are contradictory: and so far we fully agree with Dr. F. Nasse.*

When these considerations are kept in view, although we admit that the observations of Dr. Nasse, as well as others that might be quoted, show that much is yet to be done before the details of the theory of Bell, as far as the cord is concerned, are satisfactorily made out, we shall not find any thing in them to shake its grand principle,—that every filament of nervous matter has its own specific endowment, and is incapable of performing functions to which other filaments are subservient.

After referring to Magendie's experiments, which seem to prove the incapacity of stimuli, applied to the detached roots of the nerves, to produce motions, Dr. Nasse relates a curious experiment, performed with the view of determining how far the mental functions of the spinal cord are dependent on the integrity of its parts.

"I divided the spinal cord of a young dog immediately below the last dorsal vertebra, and having detached a string of the posterior fasciculus from the rest, leaving it connected at its inferior part with the lumbar portion of the cord, I placed it on a glass plate, and submitted it to the influence of a galvanic pile, composed of six pairs of plates, an inch and a half in diameter, in such a manner as that the electric fluid should touch only the detached portion. I then removed the detached string, and treated a similar portion of the anterior fasciculus in the same way. On neither occasions did the slightest motion appear. On the other hand, motions of the haunch and hind legs took place when the wires were applied to the uninjured part of the lumbar division of the cord."

It appears to us, however, that the previous observations of Dr. N. on the fallacies attending experiments on the functions of the spinal cord are particularly applicable in this instance; and that he could scarcely expect that irritation of the detached part of the anterior fasciculus should produce what would at any rate be a feeble effect, after the rough treatment to which the posterior fasciculus had been previously subjected.

The difficulty of ascertaining the influence of partial injuries of the

* Vide Müller's Physiology, vol. i. pp. 629 and 794.

spinal cord on its mental functions, has given rise to much difference of opinion amongst physiologists. As already stated, it is much easier to determine the loss of its motive power than the deprivation of its sensibility. The cases related by Magendie, Velpeau, Ollivier, and Dr. Nasse present contradictory results with regard to the relation of the grey matter with these functions; and our author thus concludes his account of them:

"If we compare the cases in which the manifestations of sensation and voluntary motion ceased, although some portion of the spinal cord remained uninjured, with those cases in which, under the same circumstances, the power of motion at least was retained, we are forced to allow that there must be some parts of this organ of more importance than others to the maintenance of its mental relations. Where these parts (which are still to be discovered,) remain entire up to the period of death, and then rupture, we can conceive how the observers of such cases are led to believe that sensibility and mobility may exist after complete separation of the spinal cord. This appears to be the probable explanation of the case mentioned by Desault, in which the patient is stated to have retained the power of voluntary motion in his limbs, after complete laceration of the spinal cord in the dorsal region."

The relation of the *different regions* of the spinal cord to the sensitive and motive power of the parts receiving their nervous supply from each of these regions, is the subject next considered by Dr. Nasse, and forms one of the most interesting portions of his essay. Although a superficial enquiry might lead us to suppose that each region of the cord governs, both in health and disease, the functions of the peculiar nerves to which it gives origin, more extended observation teaches that this is by no means the whole truth, and that there is so intimate a connexion between its different portions as considerably to modify our inferences from physiological and pathological phenomena. When the injury or disease of the cervical portion of the cord is not so great as speedily to destroy life, it sometimes happens that loss of sensibility, or of the power of voluntary motion, or of both, is limited to the parts which receive their nerves from this region. This is by no means, however, the general rule; for it most frequently happens that organs supplied with nerves from portions of the cord below this region share in the paralysis. Cases also occur in which the degeneration or injury has been confined to the cervical region; and yet the lesion of function affects, not the parts which derive their nervous supply from this portion of the cord, but those which receive nerves from the dorsal or even the lumbar vertebræ. Instances of this kind are related by Pott, Sir E. Home, Boyer, Abercrombie, &c.; and Dr. Nasse mentions one which came under his own observation. It not unfrequently happens also that disease of the cord in the dorsal region affects parts receiving their nerves above this division. Paralysis of one or both arms is a common result of disease in the dorsal vertebræ; and Legallois has seen destruction of the lumbar portion of the cord in rabbits twenty days old, quickly followed by failure of the nervous energy of the cervical and dorsal portions.

"Physiologically considered, the foregoing observations seem to prove that, with respect to its relations to sensibility and voluntary motion, the vital unity of the cord, the combination of its various parts to form one whole, should be taken into account more than is generally done. There are certainly relations which belong to one of these parts more than another; each portion, however, depends upon the influence of the rest for its vital manifestations, so that, in this point of view, the cord appears to bear the same relation to its component parts as the brain."

This we think a just and important observation. The vital unity of the spinal cord is less apparent in the relations between both sides of the organ, than in those which occur between different parts of the same side, as was long since proved by the experiments of Galen. In a few cases, division of one side of the cord has produced a corresponding degree of influence on the uninjured side; although separation of the two halves of the cord by a longitudinal incision appears, from the experiments of Dr. Nasse, to have produced very little appreciable disturbance of motion. Where paralysis has arisen from the pressure of a tumour on one side of the cord, both sides have been implicated, but one side more than the other. Whether disease strictly confined to one side of the cord can in any case produce symptoms referred to the opposite side, remains to be proved.

We may next follow our author in the enquiry into the influence which the spinal cord, as an organ having such important relations to motion and sensation, may exercise on the brain. Two circumstances observed in cases of spinal disease first claim our attention: first, the loss of voice, noticed by Abercrombie; and, secondly, the gradual loss of sight frequently occurring in tabes dorsalis. The first phenomenon was observed by Chausset to follow division of the spinal cord in dogs, between the superior vertebræ; and Bischoff has shown that it always follows section of the roots of the spinal accessory nerve. Dr. Nasse found that, in cats and rabbits, division of the spinal cord in the dorsal region seemed to be followed by loss of voice in a few hours after the operation, although respiration continued. He could not distinctly ascertain that loss of sight resulted in any instance; and he is therefore inclined to attribute this occurrence, when apparently resulting from tabes dorsalis, to the influence of the spinal cord on the abdominal ganglia; and he justly appeals, in support of this explanation, to the relation, so familiar to every practitioner, between deranged states of the digestive system and disordered function of the organs of vision.

The observations of Esquirol and Ollivier on the condition of the spinal cord in cases of epilepsy, show that this organ may exercise a considerable influence on the brain. Its effect on the mental functions is also seen in cases of pressure on the cord from dislocation of the vertebræ in the cervical region. "In such cases," says Dr. Nasse, "the patient generally receives the assurance of approaching death with tranquillity, and even gaiety: indeed, this indifference about life is observed so constantly, that it may be looked upon as a general rule. Hence it would appear that, in the ordinary condition of both organs, the spinal cord exercises an influence over the brain; not, however, such as is necessary for the performance of its intellectual functions." We have ourselves seen an instance of injury of the spinal cord by fracture of the sixth and seventh cervical vertebræ, where there was no aberration of mind during the first few hours after the accident; and we have also known a case of complete loss of voluntary power over every organ below the upper part of the neck, in which recovery gradually took place, without disorder of the intellect at any period of the complaint. The inference drawn from such cases, however, can scarcely be regarded as free from objection.

In treating of the relation of the spinal cord to respiration as a voluntary act, Dr. Nasse states his objections to what he considers the weakest part of Sir C. Bell's theory respecting this function. The statements of Bell as to the origin of the respiratory nerves from the sides of the medulla oblongata and spinal cord by single roots, and the absence of ganglia upon them, are not regarded by Dr. N. as correct; and he brings forward cases of injury and disease affecting the lateral columns, in which the respiratory movements were not impaired. He does not particularly mention, however, what has always appeared to us one of the strongest objections to the theory,—that many nerves unconnected with respiration, particularly those of the axillary plexus, have always been described by anatomists as connected with the lateral columns of the cord, in the same degree as are the phrenic or the intercostal nerves.

In his observations on the dependence of the respiratory movements on the will, Dr. N. appears to us to have misunderstood Sir C. Bell's meaning; supposing him to maintain that respiration is strictly an involuntary act, as if it were managed on the same principle as circulation. But we think, that Sir Charles only asserts that the function is put under the guidance of "a sensibility more certain in its effects than the will;" in which he is clearly right. On the other hand, Dr. Nasse is certainly justified in the assertion that the nerves and muscles concerned in respiration are strictly under the dominion of the will; but we think that he goes far beyond the truth in maintaining that their necessary and constant actions are produced by the stimulus of volition. Much discussion with regard to the voluntary and involuntary contraction of those muscles which are placed under nervous influence, might, we think, have been avoided, if the source from whence that influence is derived had been strictly attended to. Nearly every muscle in the body which is supplied with motor nerves may be excited to action either by the will, or by an involuntary impulse arising from an impression made on the sensitive nerves, and conveyed to the cerebro-spinal axis; and, although it is a point at present keenly debated amongst physiologists whether or not this impression must amount to a *sensation* before giving rise to motive influence, it seems generally conceded that this influence is quite independent of *volition.* In regarding all the muscles which can be thus excited to action as susceptible of these two kinds of stimuli, one resulting from a physical impression, the other from a mental act, and both conveyed by the same nerves, we are led to perceive that the same muscle may be either voluntary or involuntary, according to the source and degree of the nervous influence by which it is excited; and the general character of the muscle will depend upon its usual or predominant action. This explanation applies with peculiar force to the mechanism of respiration, a function which is in itself purely organic, and therefore involuntary, and which in the lowest animals, as in vegetables, requires no effort for its constant performance. Though, therefore, we regard the interchange which occurs between the air and the blood as an act of the same character with the processes of nutrition and secretion in other parts, it requires for its continuance a series of mechanical changes, for which a muscular apparatus is adapted; and, although these changes are not placed beyond the control of the will, as are those *immediately* concerned in the maintenance of organic life, yet they do not appear to us to be dependent upon it. We agree with Sir C. Bell, therefore, in regarding the ordinary movements of respiration as produced by a nervous stimulus distinct from that excited by the will, (although we do not conceive that the motive influence is conveyed by different nerves;) and the muscles concerned in them, though partly under the control of volition, are compelled to exercise their functions without any lengthened intermission, by the strength and constancy of this stimulus. Other muscles, however, as those which raise the scapula, are usually subject to the will, and may be regarded as strictly voluntary, though sometimes involuntarily called upon to assist in the function of respiration, when the stimulus is excessive; but we have been ourselves acquainted with cases in which these muscles, when prevented by paralysis from responding to the influence of volition, could still be called into action by a strong stimulus originating in the respiratory organs. We may refer also to several interesting cases mentioned by Sir C. Bell, as fully proving, to our minds, that the respiratory stimulus is distinct from that of the will, and sometimes opposed to it. Some observations on instinctive actions in general, which are introduced by our author in illustration of his views on the subject of respiration, would detain us too long, were we to enter upon the consideration of them; and we, therefore, pass on to the succeeding portions of his essay.

That the desire for food does not depend on the connexion between the digestive system and the brain, through the medium of the vagus, appears to Dr. Nasse to be proved by its continuance after division of these nerves. It seems to be equally unconnected with the integrity of the spinal cord, and even remains unimpaired, when, in addition to division of the latter, the pneumo-gastric nerves are also divided. We much doubt, however, whether the experiments on animals, on which Dr. Nasse relies, will warrant this inference. It is true that dogs and rabbits will eat heartily after this operation has been performed, but is it for nothing but the purpose of satisfying hunger? The experiments of Brâchet lead to a different conclusion; and we are inclined to agree with him in believing that it is by the sensitive portion of the par vagum that the impression is conveyed to the sensorium by which the sensation of hunger is excited.

From the consideration of the mental relations of the spinal cord, we turn now to examine those which are more strictly physiological. Dr. Nasse has treated this part of the subject with his usual ability; and, although doubtful whether the distinction of the mental and physiological functions of this organ can be strictly observed, we look forwards with interest to the continuance of his investigations. He considers the spinal cord with reference to its influence on muscular contractility, on the circulating system, on the peristaltic motion of the intestines, on the functions of the rectum and bladder, on the contractions of the uterus, on erection and seminal emission, on animal temperature, on nutrition and secretion, and on local and general vitality.

The various experimenters who have proved that paralysed limbs retain their contractile power for some time, have made no attempts to ascertain what is the exact share which the spinal cord has in the maintenance of muscular irritability under such circumstances. The investigations of Dr. Nasse, with the view of determining this point, accord fully with the views of those who regard this property as implanted in the muscular fibre itself, and as independent of nervous influence. The purpose of his first experiment was to ascertain the relative power of the stimulus excited by the will and that of galvanism in exciting muscular contraction; and he found that the leg of a rabbit, which could raise by a voluntary effort a weight of four pounds, even after the lumbar portion of the spinal cord had been laid bare, was scarcely excited to lift it by the transmission of a smart electric discharge through the inferior portion of the cord, when separated from the rest.

Although the motions observed in limbs for some time after their nervous connexions are divided, and the temporary increase of irritability under the same circumstances, seem to prove that this property is independent of the large nervous masses, yet it appears, from the experiments of Müller and those of Dr. Nasse, that paralysed muscles lose this property after a certain time. It becomes an important question, therefore, whether its abolition is the immediate result of the interruption of the influence of the spinal cord, or is indirectly caused by the impairment of the nutritive processes. To ascertain the truth on this subject, Dr. Nasse divided the spinal cord in frogs (during the winter season,) immediately below the occiput, then cut the nervous trunks passing to one of the hind legs from their connexions with the spinal cord close to the vertebræ, and exposed both hind legs to the influence of a galvanic apparatus, composed alternately of plates of copper, iron, tin, lead, and zinc, the chain being completed with silver wire. By these means he found that the irritability of the sound limb was usually less than that of the one whose nerves had been divided, (a greater stimulus being required to produce the same effect,) and that its duration was also inferior; and the last result was still more evident when the head was cut off, instead of the spinal cord being divided. The results of these experiments lead, therefore, to the belief that the spinal cord possesses scarcely any immediate influence on the maintenance of irritability; and consequently favour the supposition that the section of the nerves derived from the spinal cord abolishes this property by means of the gradual changes which it produces on the nutrition of the nerves and muscles. They appear to us to be in strict accordance with those of Dr. Wilson Philip, and with the more decisive experiments of Dr. Reid, in which it appeared that, after the irritability of muscles had been exhausted by irritation, it could be perfectly recovered, although their nerves were cut.*

The controversy respecting the influence which the spinal cord exercises over the action of the heart, in which Dr. W. Philip and Le Gallois occupied the most prominent position on opposite sides, must be still fresh in the memory of several of our readers. We believe that the doctrine put forth by Dr. Nasse, about the period of the controversy, and afterwards supported by his experiments, is regarded by many as correct; viz. that the influence of the spinal cord is an important co-operating cause in the circulation, but that it is by no means the sole agent.

Regarding the influence of the spinal cord on the peristaltic motion of the intestines, the next subject treated of by our author, it is difficult to arrive at any unexceptionable conclusions. So many complicating

* Transactions of the British Association, vol. iii., pp. 671-4.

circumstances influence this action, that inferences, either from the results of experiments or of disease, must necessarily be open to much fallacy. After the spinal cord in the dorsal region of a rabbit was divided, Dr. N. found that a drop of croton oil produced no alvine evacuation. Brâchet obtained a contrary effect; but Schröder Van der Kolk and Fock have since found that croton oil has no effect on healthy rabbits.* As to the effects of spinal paralysis in the human subject, there is equal difference of opinion; but those who have observed the constipation which so frequently follows injuries of the lower extremities, requiring absolute rest in the supine posture, would not feel much difficulty in attributing to a similar cause the inactivity of the bowels which sometimes accompanies paraplegic affections. Gölis states that, in inflammation of the spine in children, extremely small doses of calomel unload the bowels; and this, if there be no complication in play, might be a valuable diagnostic between inflammation of the brain and spine.

That the spinal cord has an influence over the functions of the bladder is beyond doubt; but it remains to be explained why, in some cases of spinal disease, we find retention; in others, involuntary discharge of urine. The results of experiments by Dr. Nasse, Brâchet, and others, lead to the inference that considerable injury or division of the spinal cord is generally followed by retention of urine, although a discharge frequently takes place before the death of the animal. On the other hand, there are many cases which tend to prove that incontinence is more frequently connected with slighter injuries of the spinal cord; and it is a common symptom where disease of the cord comes on gradually. In a remarkable case of paralysis, mentioned in the 21st volume of Hufeland's Journal, the patient had first retention of urine, then incontinence, and, as his health improved, he recovered the natural power of retention. After some observations on the share which the diaphragm and abdominal muscles have in emptying the bladder, Dr. Nasse observes that farther investigations are necessary to determine what influence each of the regions of the spinal cord has over incontinence of urine, as also whether the different fasciculi which compose that organ exercise different functions, corresponding to the different actions of the bladder. We would observe, however, that what is usually called retention of urine does not imply loss of power in the muscular fibres of the *bladder*, which act perfectly when a catheter is introduced. It seems to be either a tonic spasm or a loss of the power of voluntary relaxation in the *voluntary* muscles which surround and compress a portion of the urethra. On the other hand, the incontinence of urine may proceed either from loss of tone in these muscles, or, in the later stages of paraplegia, from increased stimulation of the bladder, connected with an inflamed state of its mucous membrane. These considerations, which we suspect are often overlooked, incline us to believe that the muscular fibres of the bladder itself are, like those of the intestinal canal, excited to action by the direct stimulus of the fluid contents of the viscus, independently of nervous agency; but that the associated movements which, in the usual state of the body, are connected with their contractile efforts, are analogous to those of respiration, being produced by an impression conveyed from the

* Vide Müller's Journal, 1836, p. cxl.

lining membrane of the bladder to the spinal cord, and thence acting on the motor nerves.

There is no less difficulty in ascertaining the influence of the spinal cord on the contractions of the uterus. From Brâchet's experiments, it has been concluded that the power of the uterus is destroyed by injuries of the spinal marrow, which produce complete paraplegia; but the cases adduced by him do not seem to us to prove more than that the parturient efforts are not, under such circumstances, sufficient to terminate the labour. Dr. Nasse relates an interesting case, communicated to him by Professor Succow, of Jena, in which painless labour and delivery took place during the seventh month of pregnancy, after fracture of the third and fourth cervical vertebræ by a fall, and complete paralysis of all the inferior nerves. The child was dead, and the mother died not long after delivery; her intellect, and power over the parts above the seat of the fracture, remaining unimpaired to the last moment. A case is related by the late Dr. Cheyne, in which a living child was expelled, although one-half of the body was paralysed; and a similar instance has occurred within our own knowledge. We can have no doubt, therefore, that effective uterine contraction may take place independently of any efforts or power of exerting efforts to produce voluntary muscular action.

Erections and seminal emissions, but particularly the former, are of exceedingly frequent occurrence in cases of disease or injury of the spinal cord; and, in the experiments of Segalas, D'Etchepare, and Serres, these phenomena followed injury of the spinal cord after its separation from the brain. Dr. Nasse thinks, therefore, that the arguments which have been alleged to prove a peculiar relation between the cerebellum and the organs of generation are, for the most part, equally applicable to the upper part of the spinal cord, the condition of which has not been sufficiently attended to, either in experiments or pathological observations. We believe that some of those who regard the cerebellum as the peculiar residence of the instinct of procreation are inclined to limit its seat to the vermiform processes, which entirely constitute the organ in fishes; and if these processes be regarded, as we think they may, in the light of prolongations of the medulla oblongata, the views of these individuals will nearly correspond with those of Dr. Nasse. We do not, however, feel inclined to enter at present on the discussion of the merits of this question, and shall pass on to a more practical subject.

That the temperature of paralysed parts is generally below the normal standard, is now universally admitted. From the observations of Dr. Nasse and Mr. Dundas on pathological cases, it appears that the diminution of temperature is more in proportion to the loss of sensibility than of the power of motion. But, as these observations are open to many sources of fallacy, our author endeavoured to determine the question experimentally, by dividing the anterior fasciculus on one side and the posterior on the other, the temperature being measured in small wounds of the same sides, made in similar situations on each extremity. In the first experiment, on a rabbit, there was a difference, after five hours, of half a degree (Reaumur,) in favour of the limb in which motion was paralysed; in the second, on a cat, the difference was greater, but it was found after death that the anterior fasciculus had not been completely divided. A third experiment again exhibited a trifling advantage in favour of the same limb, especially half an hour after the operation; in a fourth, the whole left half of the spinal cord was divided in a full-grown cat opposite the tenth dorsal vertebra, and no difference was noticed; which we think may have been owing to the partial interruption which the physiological functions of the cord may undergo from simple division. Dr. Nasse intends to follow up these experiments, varying them so far as to obstruct or at least diminish the afflux of blood to the paralysed parts.

Numerous observations prove that many of the fluid secretions are influenced by the conditions of the spinal cord. The following experiments were made by Dr. Nasse, to ascertain what effect division of the cord has on the secretion of bile.

"The gall-bladders of five full-grown rabbits were opened at one end, the contents received on paper, and the open end closed by a ligature; the external wound was then closed with sutures, and the spinal cord divided between two of the inferior dorsal vertebræ. For the space of four or five hours after this operation, the animals ate more or less. In three which died between the twelfth and twentieth hours, the gall-bladder contained nothing which could be looked on as bile; the first contained a reddish fluid which appeared to be diluted blood, probably resulting from the mode in which the operation (the first of the kind) was performed; the other two were nearly filled with a colourless watery fluid, which became slightly coloured and turbid on the addition of muriatic acid. In the fourth rabbit, in which the contents of the gall-bladder had been removed ten hours after the operation, and again twelve hours subsequently, the fluid found in the gall-bladder, some hours after the animal's death, was more abundant and of a yet lower colour than that which had been taken out at the former of these periods. It was also rendered more turbid by the addition of muriatic acid, and yielded a more copious precipitate with acetate of lead. In the fifth, which lived thirty hours, the gall-bladder was nearly filled with a yellow fluid yielding a copious greyish white precipitate with acetate of lead, which dissolved for the most part in alcohol, communicating to it a grass-green tinge. The difference of these results from similar experiments can be attributed only to the different degree of vital power which the animals previously possessed."

Dr. Nasse is led to believe, from the facts which he has collected, that the state of the spinal cord has more immediate influence on the processes of nutrition than that of the brain; but we do not perceive in them any real information on this subject. The last topic to which he directs our attention is the influence of the spinal cord on local and general vitality. Many observers have noticed the extraordinary liability of paralysed parts to mortification, when exposed to degrees of heat and cold which produce little or no effect on sound parts; and that the parts which suffer from pressure are sooner and more extensively attacked with gangrene in palsy than in other forms of disease. It is evident that the lowered temperature of palsied parts is one cause of the increased effect of the application of heat; but this partial explanation will not apply to the opposite change; and we believe with Dr. Nasse, that the lowered degree of vitality, implying that any increased vital action is sooner followed by depression, is also concerned in producing the difference. Dr. N. has observed that paralytic patients, in whom softening of the spinal cord has been discovered after death, remain exempt from gangrene longer than others; a fact that seems to correspond with the ordinary phenomena of softening, in which lesion of motion is much more common than lesion of sensation. He proved also by well-conducted experiments, that Legallois was right in ascribing the fatal result of removal of the lumbar portion of the spinal cord to the severe shock thus given to the general circulating system. Having divided the spinal cord in the neck of several rabbits, he cut out the lumbar portion, carefully avoiding all crushing or laceration. After the first operation, the rabbits looked around with a vigilant air, attempted to get away, and took food; but as soon as the lumbar portion of the cord was removed, they laid the head on one side, the pulsations of the heart could be heard no longer, the animal gasped for breath and died. One died a minute after the latter operation; the rest did not live four. This result is modified by the age of the animal; for a young dog lived several hours after the removal of the lumbar portion of the cord; and it also occurs as a consequence of the removal of the posterior fasciculi only. The following are his conclusions with regard to the modus operandi of this injury:

"The proximate cause of death, in cases where the lumbar portion of the cord is separated from the nerves and parts connected with it, can scarcely be anything but the sudden arrest of the motion of the blood. It is not stoppage of respiration, for the arteries contain bright red blood; perhaps there may be some unknown derangement of the composition of the blood having no connexion with coagulation. Two circumstances may diminish the operation of this cause, so dangerous to life; first, a diminished necessity for blood and respiration, as in the fœtus, in newly-born animals, and in cases in which the brain has suffered violent concussion or is wanting; and secondly, an increased degree of power of the heart and of the vital energies which otherwise influence its actions. The difference in the proportion of this power in different animals, and even in individuals of the same species, must make considerable differences in the results of cases of such partial removal of the spinal cord; a circumstance which Legallois complains of as having interfered with his investigations."

At the conclusion of the foregoing article, Dr. Nasse announces his intention of continuing his investigations on many of the points already noticed, as well as others which have been touched on slightly or not at all; among the rest, he proposes to examine the doctrines which have been recently promulgated by Dr. Marshall Hall. We need not say that we look forward with great interest to the results of his future researches.

### II. *On the Diseases of the Brain which consist merely in Functional Disturbance, particularly with Reference to Diagnosis.*

This Essay, also by the elder Nasse, is contained in the Third Fasciculus, published in 1836. As the subject is one of the highest practical importance, we shall examine at some length how far our knowledge of it is extended by the present contribution.

Every pathologist is aware that, in tracing the phenomena of functional diseases of the brain, impediments beset almost every step of his progress. The mental phenomena, which constitute the most prominent indications in many cases of cerebral disease, are regulated not merely by the condition of the brain, but in great degree by causes totally distinct from organic agency of any kind. It is unquestionable, on the other hand, that the corporeal state exercises an energetic influence over the origin and course of diseases of the mind; but our knowledge is here much too vague to allow us to hazard even a probable speculation on the organic changes which give rise to mental processes, or are instrumental to the manifestation of their results in health, and *a fortiori* in disease. There is great difficulty, therefore, in connecting by the relation of cause and effect any particular material changes in the brain with disordered mental phenomena, even when they are distinctly concurrent; and as the brain, like other organs, is liable to variations in its functional activity, giving rise to states strikingly distinct, but apparently independent of any material change, it is obvious that the difficulties to be encountered in fixing and analysing the symptoms which characterize its deranged conditions, are almost indefinitely increased. The loose terminology of writers on diseases of the brain is also a source of error in drawing inferences from their statements; they have in most instances overlooked the distinction between mediate and immediate symptoms, and have classed all, whether primary or secondary, mental or corporeal, under the same denomination, cerebral. But a headach and an incoherent expression do not stand in the same relation to the brain; the latter is not, like the former, an immediate product of the diseased organ, but depends upon the alteration which the mind undergoes in certain conditions of the body. Independent of these sources of difficulty are the endless varieties of age, sex, temperament, habit, and coexisting disease, which become so many sources of complication and obscurity.

Pathological anatomy, as at present pursued, is not likely to afford much assistance in the study of the functional diseases of the brain. The material changes of which our present modes of investigation render us cognizant, are only the occasional attendants or the ultimate products of functional disturbance; and do not stand in any fixed relation to derangements in the activity of the organ. Every pathologist is aware that symptoms not only of mental disease, but of excited or depressed nervous energy, may occur during life, without any appreciable alteration in the subsequent appearance of the tissues. But, even with these facts in view, it has been too often forgotten that disease in its primary form is usually a change of vital condition not to be detected in the dead body; and that the morbid appearances sometimes discovered are valuable only as leading us to some knowledge of those preceding vital changes on which the functional disturbance usually depends. Thus the state antecedent to most degenerations of the brain, supposed by some to be congestion, by others denominated inflammation, cannot be that which originates the symptoms; since its existence in all cases is very doubtful, and, if admitted, we cannot help supposing some previous vital change which precedes and gives rise to it, and determines the nature of the resulting effect. Our knowledge, therefore, of the nature of functional diseases of the brain is not more certain when we find them accompanied by organic changes, than when no morbid appearances can be detected. In both cases we are equally ignorant of the causes which give rise to the derangement; and our duty is therefore to observe carefully and compare cautiously individual cases of such disorders, so as to arrive, by a legitimate process of induction, at such an arrangement of the phenomena which they present and the causes from which they proceed, as may be applicable to practical purposes.

After detailing at some length the difficulties attending the investigation of functional diseases of the brain, Dr. Nasse observes that they do not prevent us from carrying the diagnosis in such cases to a considerable degree of perfection. We may be able, for example, to distinguish simple functional diseases of the brain without being able to fix the symptoms which characterize affections of its individual parts; we may distinguish between meningitis and cerebritis, and observe the difference of function which the brain exhibits according as the superficial or deeper parts of its substance are occupied by disease.

The subjects to which the present essay is devoted are as follows:—1. Increased excitement of the brain. 2. Deficient excitement of the brain. 3. Morbid increase of the excitability of the brain. 4. Diminished excitability of the brain. 5. Congestion of the brain. 6. Plethora of the brain. 7. Exsanguine state of the brain. 8. Morbid preponderance of the arterial or venous system of the brain. 9. State of the brain in concussion. Other points, particularly the effects of pressure and inflammation, will form the subject of a future communication. It will be seen that this arrangement is formed on the known or supposed causes—not the symptoms—of the disorders in question; and it seems to us a serious objection to it, that the five last-named causes are much more distinct and tangible than the four first: most of them being, as we have before intimated, the results of other less appreciable morbid changes. Moreover, as the first are in fact only inferred from the absence of any causes of the latter kinds, for the purpose of explaining the phenomena of individual cases, the last heads should have been considered first; indeed, we should have thought it better to treat of the effects of pressure and inflammation, the kinds of injury which present the most definite objects of study, before entering on any of the more obscure topics here enumerated. We must also observe, that the whole enumeration is defective, inasmuch as there are two other causes to which we have no doubt, as has been remarked in a former number, that many functional disorders of the brain may be ascribed; viz. altered nutrition of the nervous matter, (as in the case of excessive quantity of phosphorus in its composition,) and altered *quality* of the blood pervading it, as in the case of absorbed poisons or retained excretions; or, to express the same in more general terms, the author has spoken of increase and diminution in the excitability of the organ and the stimuli applied to it, without sufficiently attending to the *qualitative* alterations of which both are susceptible. We shall now consider seriatim the divisions under which Dr. Nasse's enquiries are comprehended.

1. *Increased excitement of the brain.* As the function of every organ depends upon its vital properties being excited to manifest themselves by their appropriate stimuli, it is obvious that an excess or diminution of the stimuli on the one hand, or of the vital properties on the other, will give rise to disordered action. This is undoubtedly the most philosophical mode of distributing the causes of functional disturbance in cases where we can discriminate them with any probable correctness. Thus, the state usually understood in this country by the term *irritation* may arise from increased stimulation, (as when the eye is dazzled by a brilliant light,) or from increased excitability of the organ, (as in photophobia from morbid sensibility of the retina.) This term has, however, been recently employed by some writers to designate the natural state of activity of any organ; but it would, we think, be better not to change its old signification, as the word *excitement* expresses all we require for the latter purpose. Although most physiologists are in the habit of speaking of the external influences, by which the functional manifestations of an organ are excited, as *stimuli*, Dr. Nasse objects to this application of the term, which he restricts to such influences when excessive in quantity or injurious in character, so that the action which results from them is of a morbid kind. We do not, however, see the possibility of drawing a line which will define with precision the boundaries of these classes of influences; and we prefer retaining the term *stimulus* as applicable to any agent capable of exciting a vital action in an irritable part; since the nature and degree of that action will bear a strict relation to the nature and degree of the stimulus.

We shall not at present enter upon the oft-disputed question of the nature of the functions of the brain, since it is of no consequence to the present enquiry whether the corporeal structure itself performs the processes of mind, or furnishes the conditions for their manifestation. Whichever of these two be its purpose, it is admitted that its action is the result of the effect of stimuli, whether physical or mental, upon its structure; which structure being possessed of excitability (or in other words of certain peculiar vital properties,) manifests it by the phenomena it then presents in a state of healthy excitement. But when either the stimulus or the excitability is in excess, a state of irritation is induced, which may be so trifling as scarcely to deserve being regarded as a morbid condition, or which may be fraught with the greatest injury both to the functions of the organ itself, and of the whole system with which its sympathies are so close. These are, so far as we can understand, Dr. Nasse's views on this subject; but they are rendered rather obscure by what we deem the misuse of the term Irritation which he applies to the first division of functional disturbance, to which we have given the designation of increased excitement. We trust that we shall be excused for entering into these details on a subject so trite and hacknied; but there has been in physiology and in pathology a great deal too much of that vagueness in the application of terms which retarded the progress of the exact sciences in ancient times; and there can be no doubt that correct definitions are the best foundations for any subsequent process of reasoning.

Of the form of irritation of the brain arising from increased stimulation, the causes are fully but not very discriminately enumerated by our author; the mental and bodily causes are classed together, no distinction is made between alteration in the quantity or character of the natural stimuli and the addition of others entirely new; and we find mental inquietude, application of heat or electricity, pressure, intestinal derangement, &c. all placed on the same footing. From simple irritation of the brain may arise, according to Dr. N., headach, sleeplessness, convulsions, delirium, insanity, mania, epilepsy, irregularity of pulse, and fever; also starting from sleep, irritability of temper, anxiety, and certain nervous affections. It is plain that, according to the phraseology of our author, death cannot be the result of violent irritation of the brain, as Piorry supposed, without the supervention of some other of the affections he enumerates. A common result of cerebral irritation is, that it renders other states to which it allies itself more intense and protracted; for example, the apoplectic tendency, and insanity from other causes.

Dr. Nasse enters into farther discussion as to the dependence of such a train of symptoms on the state which is strictly termed irritation of the brain.

"Experiments on animals, in which injuries of the brain appeared to be unaccompanied by pain, seem at first view to discountenance the idea that *pain* is attendant on irritation of the brain. We must, however, remember that the animals used for these experiments were generally thrown into a state of stupefaction by the opening of the skull and the effusion of blood in and upon the brain, and consequently must have been rendered less capable of exhibiting manifestations of pain."

In this and some of the following observations we think that Dr. Nasse has not sufficiently distinguished the brain proper from the enkephalon. According to the opinions usually received at present, sensibility to pain, though not an attribute of the brain proper, is the chief vital endowment of parts of the nervous matter within the head: and it is certainly true, as stated by Dr. N., that, when the irritating cause is in the brain proper, the pain sometimes remains confined to the irritated spot.

"In general, however, it extends farther within the skull. Sometimes it is felt less at the spot where the irritating body (a tumour, for instance,) lies, than in another and a distant situation. . . . Whether headach from irritation of other parts may become as intense as that which proceeds from an affection of the brain itself, is doubtful or improbable: indeed, the slight degree of headach in fevers complicated with abdominal disease enables us to distinguish them from meningitis." . . . . "Sleeplessness is another symptom which indicates disease of the whole brain. But in this state, as in others, somnolency may be combined with inability to sleep. The irritation does not permit it to terminate in sound sleep, or the patient slumbers awhile and then awakes, generally with a start." . . . "Convulsions frequently occur in children, particularly during dentition and abdominal affections. In the spasmodic attacks to which young females are subject, the brain has evidently a share, although the source of irritation lies in distant organs."

It is obvious here, again, that our author uses the term brain too vaguely, convulsions being strictly a symptom produced by an irritating cause acting (sometimes *through* the brain,) on the medulla oblongata and spinal cord.

"When delirium ceases immediately on the removal of a foreign body from the brain, it would appear that the state producing it was merely one of irritation. On the other hand, where stupor ceases, or where the pulse becomes stronger and fuller after the removal of a foreign body from the brain, we must say that the usual effect of pressure, rather than of irritation, has been produced and removed."

Dr. Nasse remarks, that the mental changes resulting from irritation of the brain are very different, according to the constitution of the individual and co-existing disease; and points out the frequency of maniacal excitement arising from derangement of the abdominal viscera, usually first showing itself by great irritability of temper. He does not think that fever, strictly speaking, can arise from simple irritation of the brain, but that the heart's action may be excited and disordered.

2. *Deficient excitement of the brain.* This state is thus characterized by our author:

"We recognize a state within the limits of health,—the state of tedium or ennui,—in which the activity of the brain becomes languid from the absence of the customary vital influences, and in which the languor exhibits itself in the glance of the eye, in the lineaments of the countenance, and, above all, in the sluggishness of the pulmonary circulation and frequent yawning. Whether, and how far, diminution of the vital influences essential to the natural state of the brain indicates disease, may be doubted. There are, however, two states which may evidently be attributed to this cause. The first of these is, where men, who have long led an active life, on withdrawing into retirement and ease, fall after some time into a state of mental apathy; the second is, where mental derangement is occasioned by fixed sorrow."

3. *Morbid increase of the excitability of the brain.* The meaning attached by Dr. Nasse to this term will be understood from our previous observations; and many of his observations on the state which it characterizes are of great value.

"Dissection affords no data to arrive at any conclusions with regard to the nature of this condition. There is no foundation for the assertion that the nervous influence is increased and exalted in morbidly susceptible parts: on the contrary, it can be shown that the nervous influence in such parts is diminished."

The symptoms which characterize increased excitability of the brain itself are headach, occurring under the ordinary vital influences; sleeplessness; vivid delirium; mania, under circumstances rendering this affection persistent; excessive sensibility of the external senses; convulsions. The absence of coincidence in these symptoms, and the predominance at one time of affections of the mind, at another of the senses, and at a third of the locomotive organs, depends most probably on the diseased state never affecting the whole brain simultaneously, and on the difference in the affected parts. These symptoms, it is true, taken in general, occur also in irritation of the brain, from increased excitement; but, as Dr. Nasse observes, the two states are demonstrably different. In increased excitability of the brain, the usual vital influences are sufficient to produce the violent and rapid actions, without any discoverable sources of irritation. His analysis of the symptoms indicative of this condition appears to us sufficiently valuable to be quoted nearly in full.

"The headach is intense, and most frequently *periodic* in its attacks; it is increased by the customary vital influences, and still more by stimuli. It increases after venesection performed under a suspicion of the existence of inflammation. The headachs under which hysterical and hypochondriac persons labour, and which cannot be referred to any existing irritation or inflammation, are of this nature. A moderate affection of this kind, connected with mental emotions, may continue, as pain; but it generally vanishes at the approach of delirium.

"The sleeplessness which is connected with this state, either lasts day and night continually, or it occurs only at night. The patient seldom lies broad awake; although his eyes may be open, he is subject to hallucinations, or is led away by a train of ungoverned thoughts. A tranquil sleep, could he but obtain it, would calm him in a few hours; but its absence is characteristic of the nature of the disease, and contributes largely to the production of delirium.

"Delirium may be termed the chief symptom of morbid excitability of the brain; the more complete its development, the more certain the presence of delirium. This delirium is vivid, intense, frequently wild. In general it comes on suddenly, and disappears with equal rapidity. It is frequently ushered in by severe headach, and is accompanied by hallucinations; and may, except when intense, be interrupted by the exhortations of friends. To produce delirium, nothing more is required, on the part of the body, than derangement of the susceptibility of the brain; but, whether this be sufficient of itself to bring on chronic insanity, is doubtful; that it can generate mania is certain, particularly when morbid sources of irritation proceed from the abdomen. In cases of this kind, where there is no plethora present, abstraction of blood is calculated rather to exasperate than to subdue the disease; the cause of irritation, whether originating in the abdomen or brain, must be first removed. Convulsions, accompanied by other symptoms, are not of such frequent occurrence in morbid excitability of the brain as derangement of ideas; but this remark does not apply to children, in whom delirium seldom occurs from this cause.

"It is of importance to the diagnosis of this condition that its symptoms observe a regular course, provided they are free from the disturbing concurrence of any foreign influence. In general they are paroxysmal, or at least present remarkable exacerbations, which occur, for the most part, in the evening or during the night."

We shall not detail the causes to which Dr. Nasse attributes this form of functional derangement, nor the diseases in the production of which it is concerned, since these will occur to most of our readers. He records a curious observation, however, which we cannot pass over,—that in fevers, (which seem to present increased excitability of the brain, as well as abnormal causes of irritation,) the feeble conceptions of idiots and simpletons sometimes become normally vivid and coherent about the period when delirium attacks other persons. We have ourselves seen some striking instances of the increase of intellectual power in habitually dull men, on the first occurrence of symptoms of insanity. The first effect of intoxicating liquors shows an analogous condition. A comparison of the various forms of disease attended with derangement of the cerebral susceptibility shows that delirium is a more constant symptom than pain, and seldom co-exists with it. In all cases the delirium is one of excitement: where it becomes stupid, there is reason to suspect that the state of the brain has changed. Tranquil sleep is the characteristic and complete crisis of morbid excitability of the brain.

It is not easy to distinguish the alterations of circulation and of secretion depending on this condition of the brain, from those which arise from other diseases co-existing with it; but, as Dr. Nasse observes, we can state with tolerable precision that the "vital turgor" is increased, (i. e. congestion occurs,) particularly in the eyes and face; that the head feels warmer, the skin warm and moist, the pulse somewhat accelerated but not hard, the urine generally of the natural appearance, and the bowels not particularly sluggish.

The phenomena which characterize the complication of morbid excitability of the brain, with its other diseased states, are next considered. Irritation of the brain supervening on such a condition gives rise, we are told, to a train of phenomena which differ materially from those of either of the constituents alone. At first the delirium, if present, becomes more intense; then suddenly follow exhaustion of the cerebral functions, derangements of the senses, stupor, and paralysis. Thus, it not unfrequently happened, when the stimulating practice in fever prevailed, that patients so treated fell suddenly, in the midst of their delirium, into a state resembling syncope. Where the brain is exhausted with reference to its subservience to the mental functions, but not with respect to its motive powers, an attack of epilepsy takes place. Where inflammation of the substance of the brain supervenes on this morbid excitability, there is generally suppression of cerebral activity rather than pain or delirium; while, on the other hand, in inflammation of the membranes, there is an exasperation of the cerebral symptoms. In all these complications, it is a matter of importance to weigh carefully the symptoms present when the cerebral disease first appeared, and before any aggravation occurred. The sudden occurrence and periodic return of violent pain during the course of the cerebral symptoms will sometimes serve to throw light upon the diagnosis, as being, in all probability, generally an indication of the supervention of morbid excitability. Delirium, from being slower in its development, affords less satisfactory grounds of distinction.

On this we shall only observe, that we apprehend it will be dangerous in practice to trust to any diagnosis indicating the dependence of such serious affections as are here mentioned on the condition of the brain to which Dr. Nasse attributes them; excepting only that which is founded on a cautious trial of remedies which are used on the supposition of derangement of the circulation being the most important part of the disease.

4. *Diminished excitability of the brain.* In this state the usual influences cease to produce due excitement of the cerebral activity.

"The brain labours under torpor, and stimuli are necessary to the production of its usual vital actions. Two conditions, bearing some analogy to this, occur within the limits of natural life: one is seen in the diminished susceptibility of the brain during sleep, the other in the blunted sensibility which characterizes advanced age. Of the existence of this state, which some have denied, there can be no doubt: for the diseases which furnish examples of it are by no means few."

Amongst the diseases mentioned by our author are some varieties of "nervous fevers," and the semi-comatose state often accompanying ulceration of the intestines in typhus fever. In some of these cases organic changes have been detected; but such cases did not present any remarkable difference of symptoms from those in which nothing unnatural was discovered.

"In some of these fevers, an alternation of stupidity and delirium is occasionally noticed. This, with other analogous alterations, seems to prove that the states of increased and diminished excitability of the brain are not so far removed from each other as their symptoms appear to indicate; and that, like other parts, it may fall into a state of oscillation, in which it is at one time morbidly independent of, and at another morbidly subject to, the agency of other parts of the nervous system."

In ascribing so much of the nervous symptoms of fever to mere alteration of the excitability of the brain, we think that Dr. Nasse has overlooked the changes which unquestionably take place in the character of its stimuli; for it can scarcely be doubted that the alterations, not merely in the quantity but in the chemical and vital qualities of the blood, on which all the actions of the brain and nerves are continually dependent, must produce a corresponding difference in the functional condition of those organs.

Dr. Nasse considers that some cases of apoplexy may also be referred to this cause; for, though recent improvements in pathological investigations have shown the rarity of the instances in which no morbid appearances are found, it is not impossible that, in some examinations, cadaveric phenomena have been mistaken for the indications of disease, and that the number of such cases is really larger than, from the experience of Rostan, Dr. Nasse, and others, it would appear to be. Although, when apoplexy occurs in connexion with evident congestion of the brain, we are in the habit of regarding the latter as the cause of the disease, we must not forget that either of these states may occur without the other, and that the congestion may itself depend upon some other alteration in the condition of the brain, to which the apoplexy is rather to be referred.

The disease described by Abercrombie, Hall, and Gooch, as occurring in children after copious diarrhœa or abstraction of blood, and occasionally without any known cause, is referred by Dr. Nasse to this form of torpor of the brain; but, where it is evidently the result of debility, we should consider it as more properly attributable to diminished flow of blood to the brain. Our author is of opinion that many forms of idiocy, mental imbecility, insanity, and even mania, are also connected with diminished excitability of the brain; and that this may be confined to some particular parts of the organ, destroying the balance of the functions of the whole, and giving rise to irregular trains of ideas, as in dreams. In such parts he imagines that congestion and degeneration of structure are more likely to occur from diminished circulation; and that a more intimate connexion than is usually suspected may thus be traced between nervous and sanguineous apoplexy.

"Besides the mental symptoms which are universally known, we observe in torpor of the brain inactivity of the voluntary muscles, feeble action of the heart, sluggishness of the intestinal canal, and absence of the natural turgor of the skin. The causes of this state of the brain are either such as lower the natural susceptibility of the brain by an immediate impression, as electricity, concussion, narcotic substances operating through the blood or nervous system, &c.; or they may depend upon influences producing excitement of the parts external to the brain. Thus, considerable injuries of the spinal cord, great excitement of the heart, of the uterus, or of the intestinal canal, may become sources of cerebral torpor. This affection never runs an acute or rapid course, except in some cases of fever, and seldom remains for any considerable length of time without producing more or less structural degeneration of the brain."

Although we by no means wish to discourage enquiries of the kind we have just been considering, it must be confessed that, in the present state of our knowledge of the functions of the brain, these investigations are peculiarly unsatisfactory. The whole of this discussion on conditions of the nervous system, which are inferred merely from alterations of its vital actions, seems to us to amount to nothing more than a formal and somewhat pedantic statement of the important truth, (familiar, we hope, to most of our readers,) that all the nervous actions may be variously deranged,—sometimes by known causes of irritation, sometimes independently of any obvious exciting cause,—without the structure of the nervous system being altered, the circulation in it deranged, or its chemical nature, or that of the blood pervading it, being perceptibly or even probably diseased. This truth is kept in view by every intelligent practitioner in this country, although perhaps seldom clothed in the form whose outlines we have just depicted; and we might place in striking contrast with the somewhat vague generalities of Dr. Nasse's essay, the practical details and judicious applications to be found in Sir B. Brodie's recent work on Local Hysteria.

5. *Congestion of the brain.* There is no presumed or established pathological condition of the brain to which so many cerebral affections have been attributed as that which is understood by this designation. Its existence is inferred not merely from the appearances discovered on dissection, but from the concurrence of cerebral symptoms with increased pulsation of the carotids, redness and turgescence of the face, and from the good effects of abstraction of blood. Dr. Nasse exposes the inconsistency of attributing to the same cause the symptoms of torpor and excitement of the brain, both of which are usually regarded as indicating increased determination of blood to that organ; and, we think, very justly states that both may be dependent on other states of the brain, in which congestion does not occur at all, or only as a consequence. Increased pulsation of the carotids he does not regard as of itself indicating unusual determination of blood to the brain; and redness of the face he considers an equally uncertain sign, since it occurs where no congestion exists. "The bodies of persons who have died by hangin show," he states, "how easily a remarkable congestion of the external parts may coincide with a non-congested state of the vessels of the brain." We believe, however, that all authorities on this subject agree in stating that venous congestion of the brain is at least equally constant with lividity of the face after this kind of death. Dr. N. considers that the benefit occasionally derived from bleeding in cases of supposed congestion is no proof of the existence of this condition, since it may have produced its effect by the removal of another and a previous cause of excitement.

"Lastly, congestion of the cerebral vessels, particularly of the veins, is frequently met with where the symptoms attributed to it had never existed. The cases in which pressure on the carotids was found to diminish the cerebral affection were not so much cases of congestion as of chronic local disease, in which the local mischief proved a stimulus to the rest of the brain, while subjected to the full impulse of blood, but ceased to be such when that impulse was diminished by pressure."

Hypertrophy of the left ventricle of the heart, and obstruction to the return of blood from the head, are said to be causes which necessarily give rise to congestion of the brain. Our author thinks, however, that this assertion requires confirmation. He believes that blood driven with unnatural force, by an hypertrophied ventricle, through open vessels, passes as readily through the brain as under ordinary circumstances. Obstruction to the return of blood from the head to the heart must tend, he admits, to produce cerebral congestion, if not obviated by the position of the body; but if the right ventricle receive a diminished quantity of blood, the left has less to expel. That obstruction to the return of blood from the head in the respiratory organs may go on to a very remarkable degree, without producing any of the phenomena of congestion, is seen, he remarks, in many instances of chronic pulmonary disease. Dr. Nasse thinks that Dr. Abercrombie has overlooked, in his observations on disturbance of the cerebral circulation, the existence of the cephalo-spinal fluid demonstrated in living animals by Magendie; the principal use of which our author supposes to be to prevent turgescence of one set of vessels from producing compression of the other, as Dr. A. supposes. "It should never be forgotten," he says, "in considering this subject, that morbid accumulation of blood always presupposes diminution of vital activity in the part in which it occurs." If he means that this diminution exists merely in the coats of the vessels of the part, we fully agree with him; and we are disposed to accord with him in the belief that the morbid state, of which it is a part, is sometimes the cause of the subsequent cerebral symptoms, rather than the accumulation of blood which may arise from it. Stieglitz, who is spoken of by Dr. N. as one of the best clinical observers of modern times, expresses it as his opinion that congestion seldom or never occurs in any organ in which there is not some other morbid process going on at the same time.

"What, then, is the state of the brain which is likely to give rise to congestion?—In the first place, irritation; numerous cases of congestion belong to this class. That increased excitability of the brain, in itself, is capable of increasing the quantity of blood circulating within the cranium, is not so easily proved. It may precede and usher in a state of congestion, but both cannot exist simultaneously. In the same way, a muscle which labours under persistent spasm, or, in other words, under exaltation of irritability, contains less blood. The assertion of Bricheteau that congestion and increase of intellectual power may coexist, is extremely doubtful. No congested organ exhibits increased power; neither the eye, nor the heart, nor the lungs. Congestion is much more likely to be attended with torpor of the brain. In examining the symptoms of apparent congestion of the brain, we should first consider how much of the existing phenomena are attributable to the conditions of the nervous matter already mentioned as resulting from irritation, whether simple or combined with increased susceptibility, from torpor, or from inflammation. Again, it should be borne in mind that, as the causes which produce congestion of the cerebral vessels may affect only individual parts, the congestion may be also limited to distinct portions of the brain. Flourens states that, in his experiments on animals, he has observed congestion of the convolutions from opium, of the tubercula quadrigemina from belladonna, of the cerebellum from alcohol, and of the medulla oblongata from nux vomica."

In examining the question whether there is such a thing as congestion of the brain without disease, Dr. N. makes some very interesting observations on the relation which *thought* bears to the vascular condition of the brain. He is of opinion that clear quiet thought does not require any increase of the quantity of blood circulating in the brain; and that, where symptoms of congestion occur during the exercise of thought, they are to be attributed, not so much to the mere intellectual exercise as to the presence of some disturbing *emotion.* We regret that our space forbids us from following our author through this part of his essay, which will afford matter of much interest to the philosophical or physiological enquirer. We would observe, however, that, while we admit that mental emotion is a much more powerful cause of excitement and disturbance of the circulation through the brain than the mere exercise of the intellect, we think there is evidence that increased flow of blood to the head, and increased nutrition of the organ, are excited by prolonged demands upon its functional activity, just as the flow of blood to the mammary gland is in proportion to the degree in which there is a call for the secretion,—a fact familiar to every physiologist; and disturbance of the cerebral circulation seems to us particularly liable to occur from much *voluntary* mental exertion, continually checking the natural trains of thought, and forcing them into other directions.

6. *Plethora of the brain.* This state is regarded by Dr. Nasse as distinct from congestion, because, along with some of the signs of unusual determination of blood to the brain, a state of health is maintained, although the individual is predisposed to attacks of disease in that organ. We do not, however, see anything novel in his observations on this subject; and we may take the present opportunity of stating that we do not find, in this part of his essay, any sufficient reason to change the opinions which we expressed in a former Number, that disordered circulation within the cranium, arising from increased impetus of arterial blood, or obstructed flow of venous blood, is frequently of itself the immediate cause of the symptoms both of irritation and of torpor. We do not say that pressure thus produced is the sole cause, or that it ever constitutes the whole pathology of the case; nor do we say that, in the absence of effusion or disorganization, any evidence of such pressure is to be obtained merely by anatomical inspection. But we see nothing to shake our belief,—grounded on the effects of pressure from other causes, on the whole history of many cases of such affections, and on the experience of the *juvantia* and *lædentia,*—that pressure produced in one or other of these ways, is *one frequent, and often the most remediable, cause* of either kind, or of the combination of both kinds, of affection of the nervous system.

7. *Exsanguine state of the brain.* The existence of this condition, termed by Dr. Nasse *oligæmy*, would appear to be by no means proved. He refers to it the symptoms of excitement which sometimes occur after great loss of blood; but he allows that many of these symptoms are probably in part occasioned by the affection of the general system, and particularly of the heart; since, in many cases of animals bled to death, no deficiency of blood is found in the vessels of the brain. Diseases of the brain from this cause occur, as Dr. Nasse states, most frequently in children. Cases illustrative of this are detailed by Abercrombie and Gooch: the latter mentions expressly the remarkable emptiness of the cerebral vessels which struck him on dissection. Cases of a similar description have been observed by Armstrong, Andral, Bright, Marshall Hall, and Dr. Nasse. It occurs less frequently in the adult, but is occasionally noticed as a result of uterine hemorrhage, and where venesection has been carried to an enormous amount. It is an almost constant attendant on hypertrophy of the brain, and is not unfrequently met with in the disease which has been termed Hydrocephalus internus: it may affect the whole brain or only individual parts. Dr. Nasse points out the importance of discrimination in practice between the states of oligæmy and congestion of the brain, many of the symptoms being so similar that it requires a knowledge of the previous history and attention to the general condition of the patient before a safe diagnosis can be given. He thinks that both states may belong to the same vital condition of the brain, and doubts whether oligæmy can be regarded as an idiopathic disease of that organ, although many symptoms are immediately attributable to it. On this subject we shall only refer to our former observations on the relation between the functions of the brain and the quantity and impetus of the blood propelled through it.

8. *Morbid predominance of the arterial or venous system of the brain.* Dr. Nasse apologises for the introduction of the terms *arteriality* and *venosity*, by stating that he found it necessary to allude to them, as they have been frequently employed in German works on insanity and cerebral diseases, to denote certain specific conditions. Diseased arteriality of the brain is said to be that state in which there is a strong determination of arterial blood to the head, causing mental excitement, and even mania. Dr. Nasse very properly shows, however, the fallacy of attributing these effects to the cause assigned; pointing out that, in the only case where an increased impetus of the blood is known to exist from causes external to the brain,—viz. in hypertrophy of the left ventricle of the heart, the symptoms are by no means those of mental excitement. Pulsation of the carotids, which has been much relied on as indicative of this state, has been observed by our author in a case of cartilaginous degeneration of the auricle, in which the quantity of arterial blood sent to the brain must have been materially diminished; and he considers such pulsation as indicating an obstructed rather than increased motion of blood in the cerebral arteries.

The chief evidence of the existence of morbid venosity of the brain rests upon the congested state of the cerebral veins frequently observed on dissection; but it is well known that this is no infallible proof of the existence of venous congestion during life. A very great variety of symptoms have been attributed by different writers to this cause; but none can, with any certainty, be connected with it. Dr. Nasse looks upon Dr. Abercrombie's opinion—that the quantity of blood circulating in the brain scarcely admits of any diminution, and that, when the quantity of arterial blood sent to it is diminished, venous congestion must take place,—as resting on an unproved assumption; and he refers to various experiments which prove that the quantity of blood in the brain may be diminished, and its place supplied by effused serous fluid. He mentions an experiment of Diekerhoff, in which the brain was found considerably drained of blood, without any serous effusion into the ventricles, giving colour to the supposition that the compensating change consisted in œdema of the substance of the brain itself, and concludes by observing that disease of the brain does not so much consist in accumulation of blood in its vessels, as in an altered vital condition of the brain itself.

9. *State of the brain in concussion.* Dr. Nasse claims this affection as belonging to the domain of medicine, because its existence does not necessarily include the occurrence of any palpable injury of the external parts, nor presume the necessity of an operation. We do not ourselves see the necessity of thus apologising for the introduction of such a topic; since, however desirable and convenient may be the divisions into which the practice of the healing art has been parcelled, there can be little question that the science of Pathology should include the consideration of all morbid conditions of the body; and it is of no abstract importance whether these conditions have arisen from the excess, want, or depravation of the natural stimuli to life, from the action of unusual agents of a mechanical, chemical, or vital nature, or from causes of a strictly mental character. The ascertained relations between causes and effects are in pathology, more perhaps than in any other science, the result of experience only; its laws are not yet sufficiently established, and of the influence of concurrent or interfering circumstances too little is known to enable us to proceed in a deductive course of reasoning, with any probability of success; and, in the investigation and classification of morbid processes, therefore, we are to proceed rather by what we learn by observation of their actual state and character, than by what we may suppose to be their efficient causes.

To return to our subject. Dr. Nasse observes, that the symptoms of simple concussion are very constant, and are therefore referrible to a common cause; but these are frequently united with those of laceration, effusion, &c., from which it may occasionally be difficult to separate them. He then enquires, "What is the essence of this state of the brain arising from concussion?" and expresses his dissent from the opinion of Sir Astley Cooper, Dr. Bright, and others, that the symptoms which follow it are due to derangement of the circulation. "The vessels and the blood, it is true, suffer in concussion; but they suffer, if not solely in consequence of the affection of the cerebral substance, at least only in combination with it." We think, however, that our author has somewhat misapprehended the opinion which he disputes: the affection of the circulation is not regarded as the cause of *all* the symptoms of concussion, but only of those which most clearly distinguish it from other morbid conditions of the nervous system. He rejects the statement of Littre, that concussion produces a collapsed state of the brain; arguing, very justly, that collapse of the brain can hardly take place, even when the skull is opened, without previous loss of blood or atrophy of its substance, and that it cannot take place in the closed skull, without having the space between it and the bones filled by some other substance. He rejects also, perhaps more hastily, Brodie's conjecture that, in concussion, changes of structure take place which cannot be detected by our senses, on two grounds: first, because he considers it a mere assumption; and, secondly, because it is contradicted by the sudden recovery of persons who have laboured under concussion; and he rests, therefore, in the simple expression of the fact that it is a derangement of the vital activity or excitability of the brain. It may, we think, be reasonably questioned whether the vital properties of any organ can be immediately impaired or destroyed by an impression made upon itself, without some change of structure; and we may also observe, that Dr. Nasse seems to have left out of view that the affection of the *brain* in concussion (which is only manifested by coma, and the delirium occasionally succeeding it,) is but a part only of the result of the concussion; the enfeebled state of the circulation and the frequent attacks of spasm, which are also consequences of the injury, are more directly referrible to the spinal cord. We shall not follow him through his account of the symptoms by which simple concussion may be distinguished from that complicated with laceration or effusion, since it does not contain any peculiarly novel observations: the subject is one of acknowledged difficulty, and every one must be aware that an experienced practitioner will generally give a more correct diagnosis in such cases than one who has derived his knowledge from the profoundest closet-study.

The main object of this very interesting essay, as our readers will have perceived, is to endeavour to substitute the doctrine of altered vital conditions for that of supposed pathological states, in the classification of diseases of the nervous system. Although the remarks which we have already introduced will have shown that we frequently dissent from the author, both on general and particular questions, we hope that the care which we have taken to present his views in a definite and correct form will not be without its effect in assisting some who are engaged in pathological observations in this country, to classify and generalize the results of their experience. We entertain a high opinion of the value of Dr. Nasse's investigations; and feel sure that his statements, though in some instances liable to objection, demand careful consideration in any systematic view of the pathology of the nervous system.

We regret that we cannot now notice the other interesting essays contained in these fasciculi. The high value, however, which we attach to investigations conducted like those already commented on, will lead us to take an early opportunity of returning to the most interesting of the subjects we at present omit; particularly when we are called upon to continue our account of the researches of the industrious authors, by the publication of additional parts of their Memoirs.

